# A Multiresolution Approach to Discrete Tomography Using DART

**DOI:** 10.1371/journal.pone.0106090

**Published:** 2014-09-05

**Authors:** Andrei Dabravolski, Kees Joost Batenburg, Jan Sijbers

**Affiliations:** 1 iMinds-Vision lab, University of Antwerp, Antwerp, Belgium; 2 Centrum Wiskunde & Informatica (CWI), Amsterdam, The Netherlands; 3 Mathematical Institute, Leiden University, Leiden, The Netherlands; Banner Alzheimer's Institute, United States of America

## Abstract

In discrete tomography, a scanned object is assumed to consist of only a few different materials. This prior knowledge can be effectively exploited by a specialized discrete reconstruction algorithm such as the Discrete Algebraic Reconstruction Technique (DART), which is capable of providing more accurate reconstructions from limited data compared to conventional reconstruction algorithms. However, like most iterative reconstruction algorithms, DART suffers from long computation times. To increase the computational efficiency as well as the reconstruction quality of DART, a multiresolution version of DART (MDART) is proposed, in which the reconstruction starts on a coarse grid with big pixel (voxel) size. The resulting reconstruction is then resampled on a finer grid and used as an initial point for a subsequent DART reconstruction. This process continues until the target pixel size is reached. Experiments show that MDART can provide a significant speed-up, reduce missing wedge artefacts and improve feature reconstruction in the object compared with DART within the same time, making its use with large datasets more feasible.

## Introduction

Computed tomography (CT) is a non-invasive imaging technique which is based on reconstruction of an object from a series of projection images. CT has applications on all scales, ranging from 3D imaging of nanomaterials by electron microscopy to the reconstruction of electron-density maps of the solar corona [2], [3]. In many of these applications, it is highly desirable to reduce the number of projections taken. In materials science, for example, reducing the number of acquired projections leads to faster imaging which allows to increase the time resolution to study the evolution of structural changes in materials induced by stress or temperature [4]. In electron tomography, the number of projections is kept low either to limit the acquisition time or because the electron beam may damage the sample [5].

Unfortunately, a low number of acquired projections leads to artefacts in the image reconstruction. Indeed, analytical reconstruction algorithms, such as Filtered Back Projection (FBP) [6], require a large number of projections acquired from a full angular range to obtain reconstructions of acceptable quality. Iterative reconstruction algorithms, such as the Simultaneous Iterative Reconstruction Technique (SIRT) [7], allow to incorporate prior knowledge about the object into the reconstruction such that high quality reconstructions can be obtained from even a low number of projections. Various forms of prior knowledge about the object can be employed. Sparsity of image derivative magnitude is used in a total-variation (TV) minimization algorithm to address few-view, limited-angle and bad-bin reconstruction problems [8]. Alternatively, information about the edges of the object is shown to improve the reconstruction quality in case of limited data problems [9]. Finally, prior knowledge about the number of materials has also been shown to yield accurate reconstructions from a small number of projections, which is the domain of discrete tomography [10].

Recently, a practical algorithm for discrete tomography, the Discrete Algebraic Reconstruction Technique (DART), was introduced, which is able to produce high quality reconstructions, even for large datasets [1]. Meanwhile, DART or variations of DART [11]–[14] have been successfully applied in electron tomography [2], [15], micro-CT [16], [17] and magnetic resonance imaging (MRI) [18]. However, being an iterative reconstruction algorithm, DART suffers from long computation times, which limits its use for in applications where computation time is important.

To decrease computation time or, alternatively, improve reconstruction quality achieved in a certain computation time, a new approach is proposed in which the available projection data is first reconstructed using DART on a coarse grid. The obtained reconstruction is then resampled on a grid with smaller pixels and used as a starting point for a subsequent DART reconstruction. This process is iteratively repeated until the target pixel size is reached. The proposed approach can extend the area of applicability of DART, allowing its application to large experimental datasets.

## Motivation and approach

We will now briefly outline the basic concepts of the DART algorithm [1], after which the extension to MDART is described.

**Figure 1:**
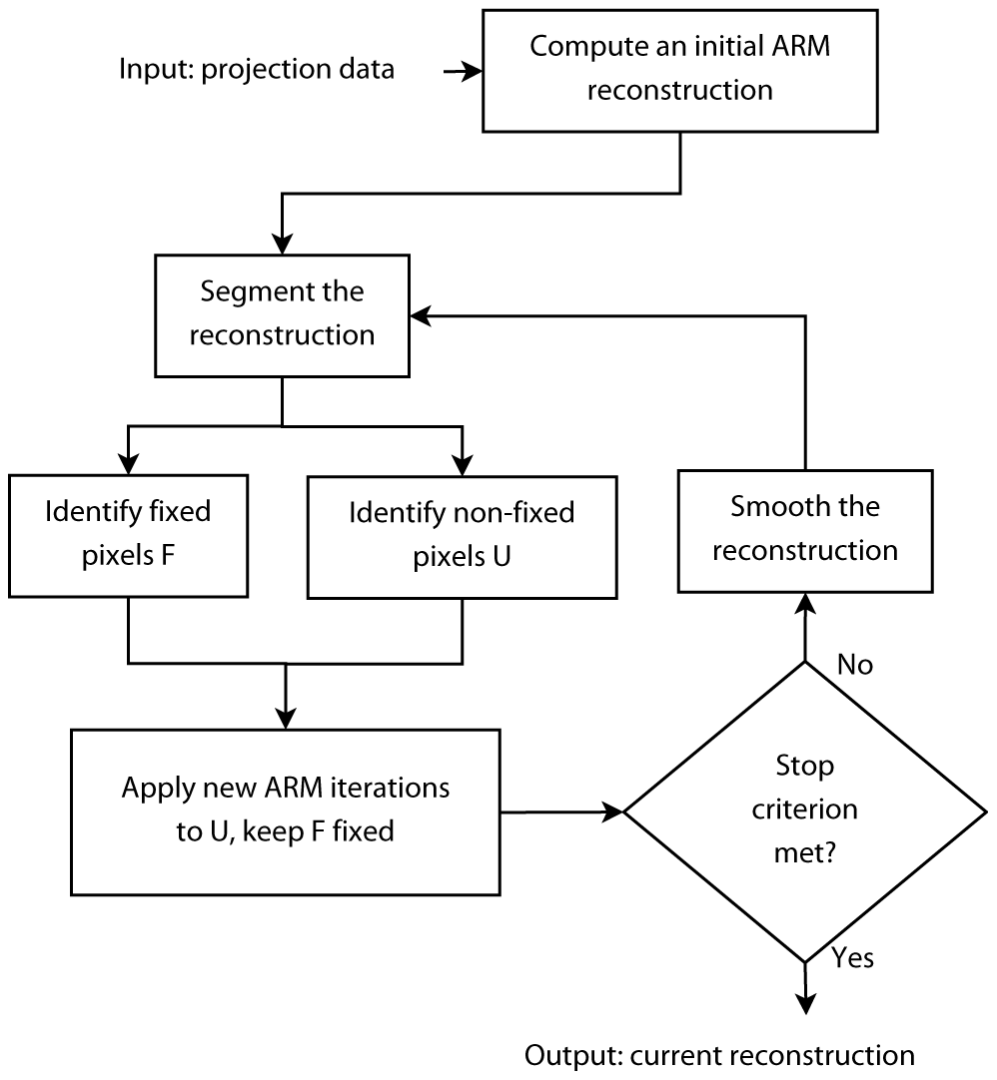
Flow chart of DART [1].

A flow chart of DART is shown in Fig. 1. The algorithm starts by calculating an initial reconstruction using an algebraic reconstruction method (ARM). This reconstruction is then segmented. Usually, only the pixels close to the object boundary can be misclassified whereas the confidence in the classification of the interior of the object and background pixels located far from the object boundary is high. Therefore all pixels are assigned to either fixed (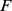) or non-fixed (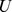) pixel sets. The non-fixed pixel set 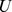 contains all boundary pixels, i. e. pixels having at least one adjacent pixel with a different grey level. A randomly chosen fraction of non-boundary pixels is also added to the set of non-fixed pixels to allow the formation of new boundaries. The remaining pixels form the fixed pixel set 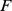. Next, several ARM iterations are performed for the non-fixed pixels while keeping the values in the fixed pixels unchanged. After that, a termination criterion is checked (examples of termination criteria are given later in this Section). If the criterion is not met, the entire reconstruction is smoothed, finishing one DART iteration. The process is iteratively repeated until a specified convergence criterion is met.

Any iterative reconstruction algorithm can be used as the ARM. Throughout the paper, SIRT [7] is used as the ARM, which is formulated as follows. Let 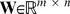 be a projection matrix and let 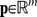 denote a measured projection data. Denoting an unknown image with 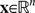, we can formulate the reconstruction problem as 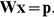
(1)The update expression for SIRT is given by [7]

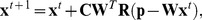
(2)where 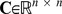 and 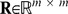 are diagonal matrices with 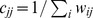 and 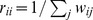.

**Figure 2:**
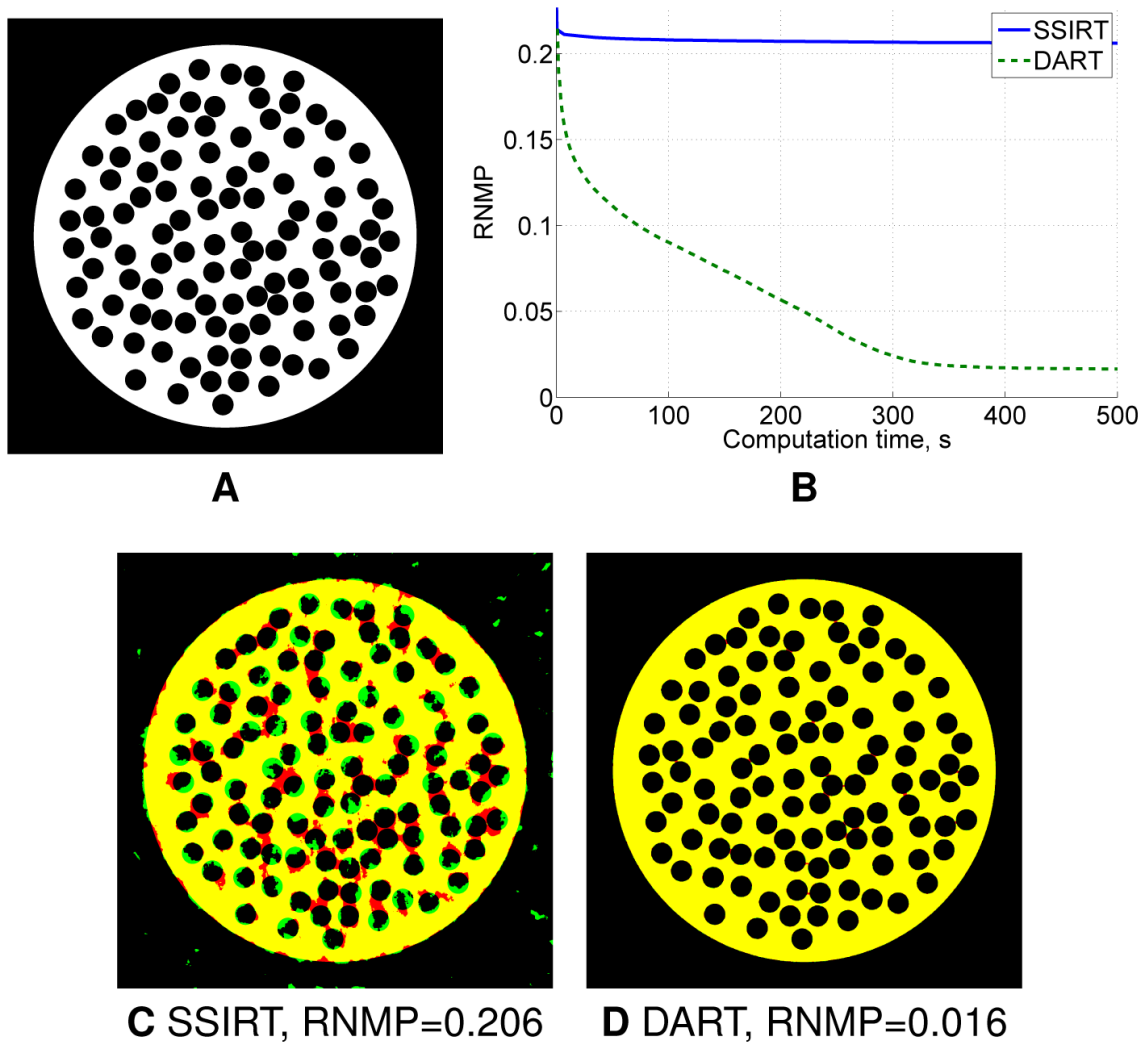
Example illustrating slow convergence of DART for some datasets. Phantom, 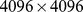 pixels size, with holes of radius 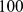 pixels (A) and RNMP as a function of the computation time for the reconstruction of this phantom using SSIRT and DART from 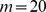 projections (B). Error images for SSIRT (C) and DART (D) reconstructions after 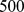 s iteration time. Red and green in the error images correspond to misclassified background and object pixels, respectively, black and yellow represent correctly classified background and object pixels, respectively.

While DART has shown its efficacy in reconstruction of micro-CT [17] and electron tomography [2], [15] datasets, in some cases DART can suffer from slow convergence, leading to long computation times required to find a practically acceptable reconstruction. Figure 2B illustrates one of such cases, where DART is capable of providing an accurate reconstruction only after a long iteration process. For the same phantom, Segmented SIRT (SSIRT) converges rapidly, though yielding a reconstruction of a poor quality (Fig. 2) (the definition of the *relative number of misclassified pixels* (RNMP) and a detailed description of the experimental conditions are given in the following section). Such behaviour of DART is explained by a highly inaccurate initial ARM reconstruction. Being calculated from only a few projections, the initial reconstruction often contains strong artefacts which then require many DART iterations in order to reduce these artefacts. Note that although the initial reconstruction has a certain influence on the convergence of DART, it does not determine the resulting reconstruction completely. Therefore, improving the initial reconstruction will lead to faster convergence and smaller computation time or to more accurate reconstructions after a fixed computation time.

In [15], applying masking during the computation of the initial SIRT reconstruction significantly reduced the missing wedge artefacts in the initial reconstruction and allowed to improve the resulting DART reconstruction. This improvement was attributed to a better estimation of grey values used in DART as those grey values were calculated from the initial reconstruction. While inaccurate grey values may indeed result in inferior quality of the DART reconstructions, even correct grey values do not guarantee fast and accurate reconstructions simultaneously (Fig. 2).

**Figure 3:**
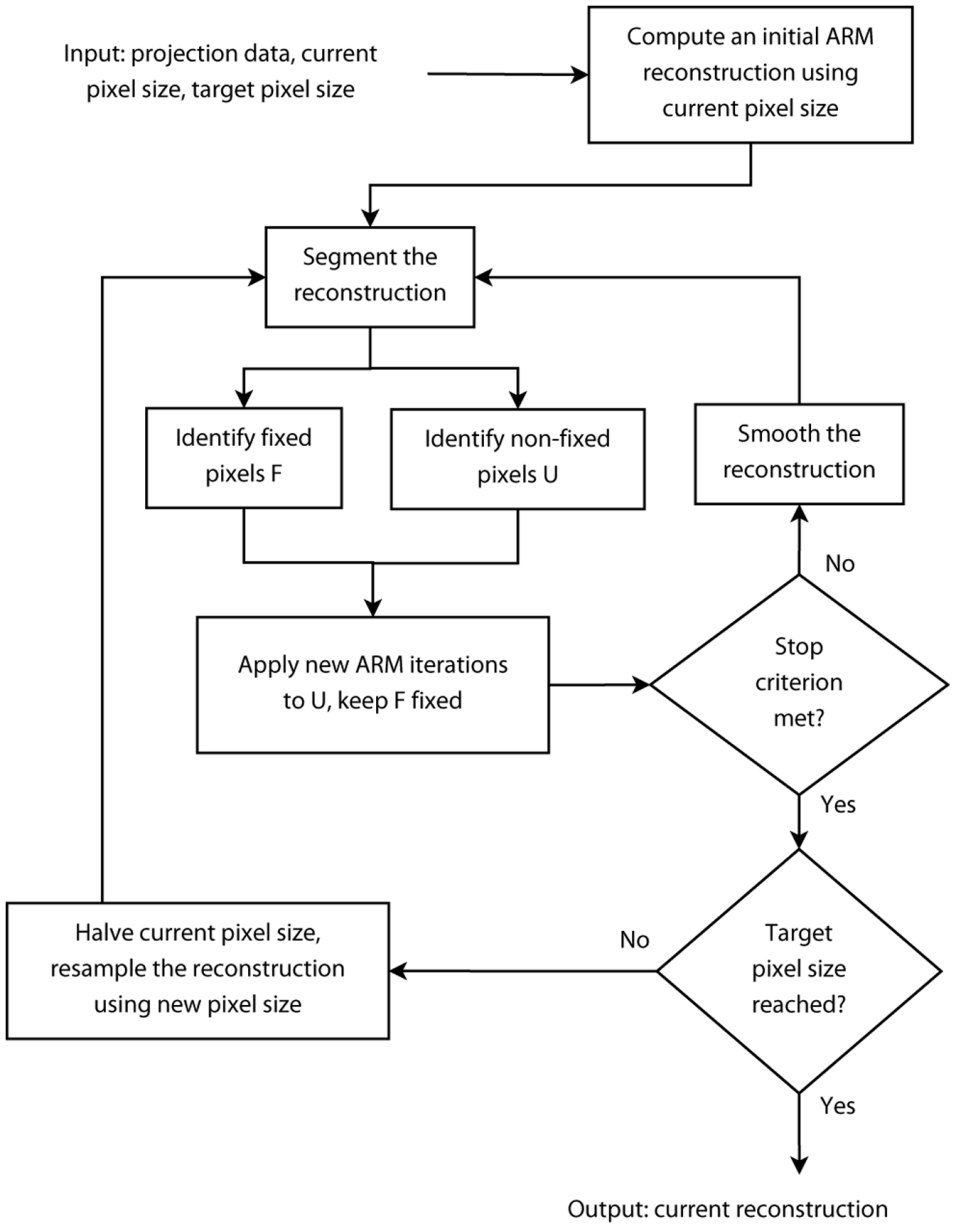
Flow chart of the MDART algorithm.

The idea of the proposed multiresolution approach (MDART) is to first start a DART reconstruction on a coarse reconstruction grid and then use the resampled resulting reconstruction as a starting point for a subsequent reconstruction on a finer grid (Fig. 3). The use of coarser grids makes the reconstruction problem less ill-posed as the number of unknowns decreases and the number of equations remains the same. This allows to compute a good estimation of the object and then improve it on finer grids to reveal finer structures which cannot be reconstructed on the initial coarse grid.

Since DART, and hence MDART, is a heuristic algorithm, there is no formal definition of the conditions which guarantee the convergence of the reconstruction process. The following termination criteria can be used in practice:

- a certain *number of iterations* are performed;
- the *relative number of modified pixels* is smaller than a given threshold. If only a few pixels change their values during the iteration, the object is mainly reconstructed;
- the difference in the *projection distance* (Eq. (3)) between the reconstructions after two consecutive iterations is smaller than a given threshold. This means that the reconstruction stops improving.

The projection distance for a reconstruction 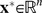 is defined as
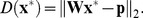
(3)


In our experiments, the modified projection distance criterion was used: iterations were stopped if the criterion held for three consecutive iterations.

**Figure 4:**
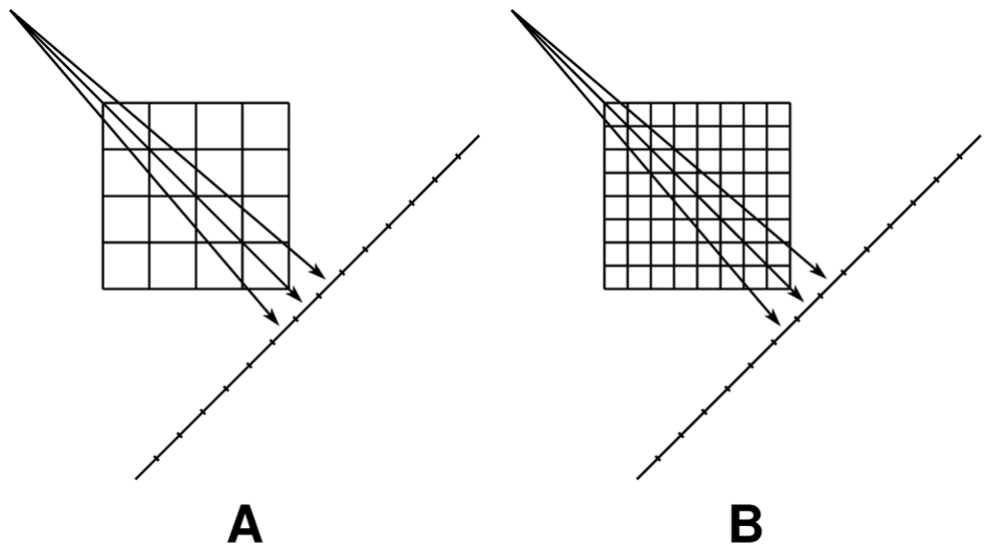
Projection geometry and reconstruction grids used by MDART 2. The coarse reconstruction grid (A) and the target reconstruction grid (B).

Let MDART 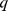 denote the multiresolution DART algorithm which operates on 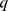 reconstruction grids or, alternatively, performs 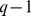 switchings to a finer reconstruction grid, in which the pixel size is halved. This algorithm starts from the pixel size which is 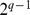 times bigger than the target pixel size. Note that MDART 1 is identical to the conventional DART. Figure 4 illustrates these concepts showing the reconstruction grids and the projection geometry for MDART 2.

## Experiments

### Noiseless simulations

A number of simulation experiments were run using phantom images to demonstrate the proposed approach. In all simulation experiments, the size of the phantoms was 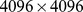 pixels while reconstructions were performed on a 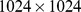 reconstruction grid to reduce the effect of the pixelation on the reconstructions. A number of 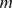 equiangular fan-beam projections were computed from the original phantoms using Joseph's projection method [19]. A detector with 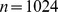 elements was used. All experiments presented in the paper were implemented using the ASTRA toolbox [20] where GPU acceleration was used extensively [21]. A desktop PC equipped with an Intel Core i7 930 processor, 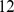 GiB of RAM and NVIDIA GeForce GTX 285 graphics card was used for computations.

Four reconstruction algorithms were compared:

- Segmented SIRT (**SSIRT**). The well known SIRT reconstruction algorithm [7] was used to calculate the reconstructions which were then segmented using a global threshold for a fair comparison.
- **DART** [1]. An initial reconstruction was calculated using 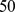 SIRT iterations; 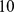 SIRT iterations were applied to the non-fixed pixels during each DART iteration.
- **MDART 2** and **MDART 4**. All parameters of the underlying DART algorithm were identical to the ones described above. Reconstruction resampling was performed using the bilinear interpolation.

Correct grey values and a global threshold were used in the simulation experiments. All participating algorithms were stopped after a certain iteration time. The quality of the reconstructions was assessed by calculating the *relative number of misclassified pixels* (RNMP) according to 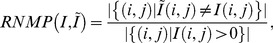
(4)where 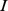 is the original phantom and 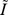 denotes the reconstruction resampled on the same grid as 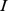 using the nearest-neighbour interpolation.

**Figure 5:**
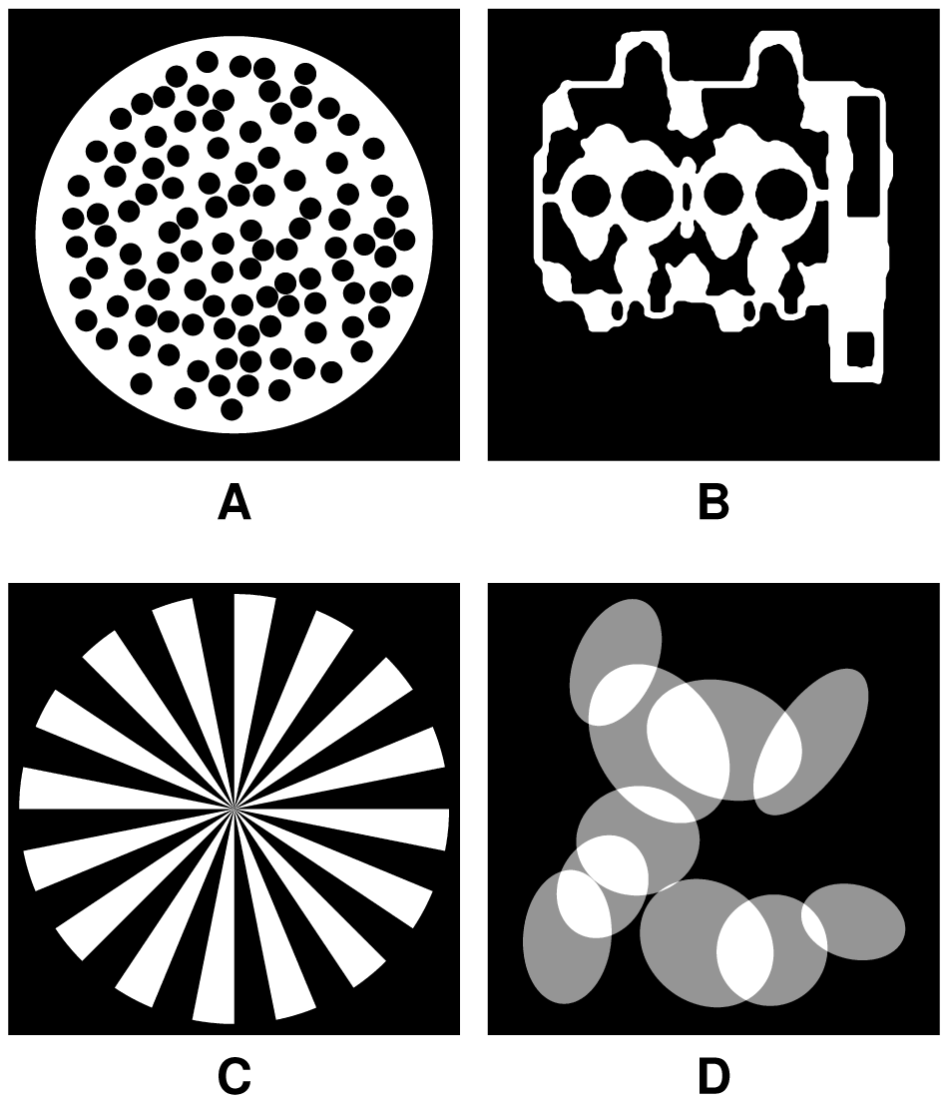
Phantoms 1–4 (A–D), 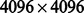 pixels.

In the first series of experiments, four phantom images (Fig. 5) were used. Phantom 1 (Fig. 5A) is a disk with a number of holes of radius 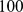 pixels. It is identical to the phantom used in the previous section (Fig. 2A). Phantom 2 (Fig. 5B) represents a cylinder head of an internal combustion engine, Phantom 3 (Fig. 5C) is a Siemens star-like phantom, Phantom 4 (Fig. 5D) consists of a number of intersecting ellipses and has three grey values, whereas the former three phantoms are binary. From these phantoms, a number 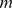 equiangular projections were computed. These projections were then reconstructed using the SSIRT, DART, and MDART.

**Figure 6:**
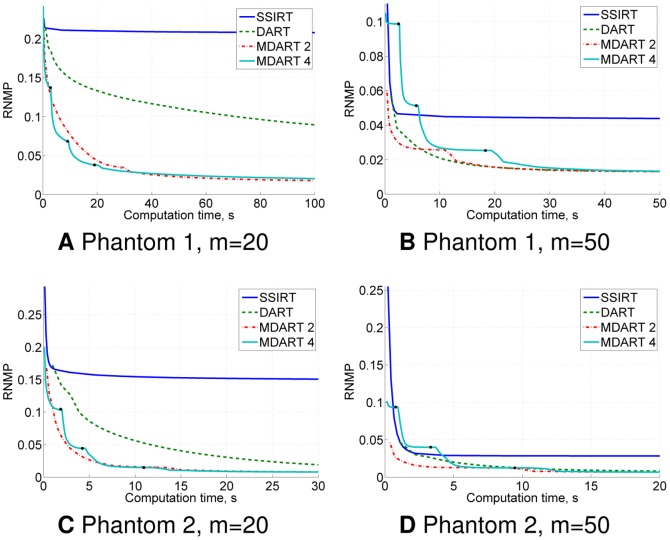
Noiseless simulation results for Phantoms 1–2. RNMP as a function of the computation time for the reconstructions of Phantoms 1–2 (Figs. 5A and 5B) from 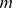 projections (A–D). Black and grey points on the MDART curves mark the moments of switching to a finer reconstruction grid.

**Figure 7:**
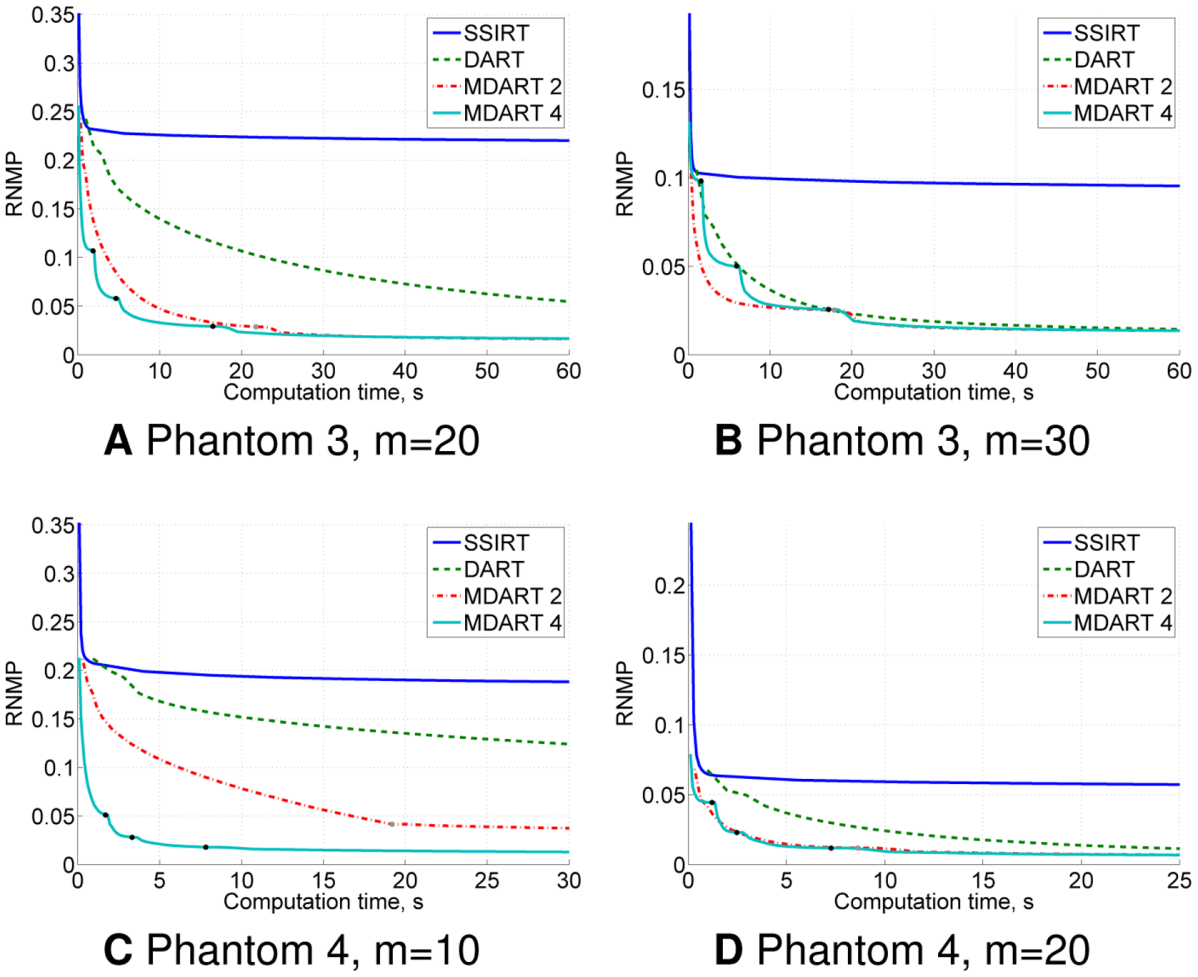
Noiseless simulation results for Phantoms 3–4. RNMP as a function of the computation time for the reconstructions of Phantoms 3–4 (Figs. 5C and 5D) from 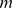 projections (A–D). Black and grey points on the MDART curves mark the moments of switching to a finer reconstruction grid.

The obtained results are shown in Figs. 6 and 7, which suggest that MDART can provide significantly better reconstruction quality in only a fraction of computation time compared to SSIRT and DART, especially when there are only a few projections available.

**Figure 8:**
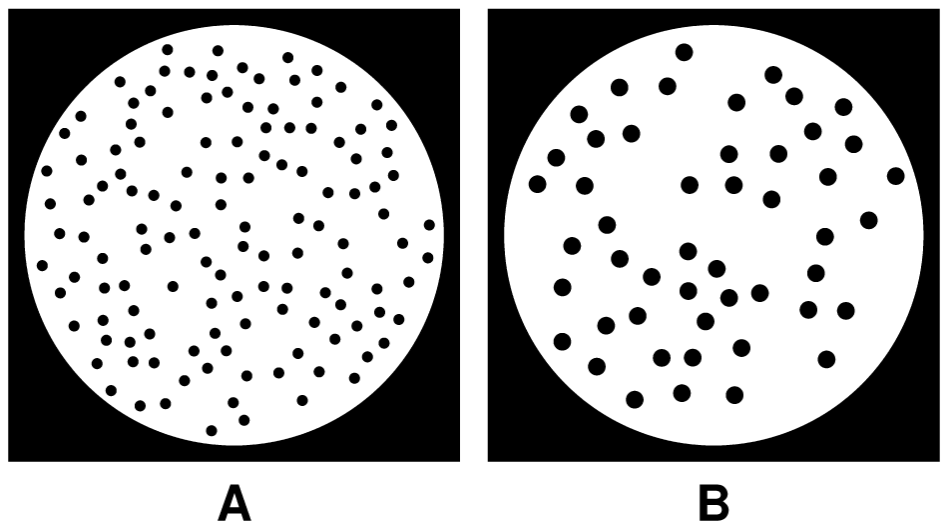
Examples of the phantoms, 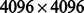 pixels size, with holes of radius 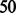 (A) and 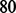 (B) pixels.

For the second series of experiments, a number of phantoms were used, each consisting of a disk with randomly placed circular holes of a particular size (Fig. 8). Three phantoms were created for each hole size. For these phantoms, projections from complete and from the limited angular ranges were computed in order to evaluate the applicability of the proposed approach for objects with features of various size and for the datasets with the missing wedge.

**Figure 9:**
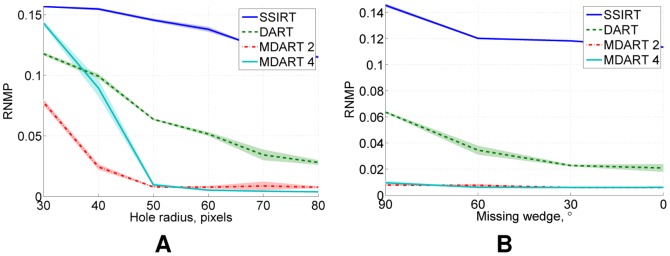
Noiseless simulation results for the phantoms with holes. RNMP for the reconstructions of the phantoms with various hole sizes from 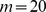 projections after 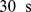 iteration time: (A) as a function of the hole radius for the 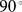 missing wedge and (B) as a function of the missing wedge for the phantoms with the hole radius of 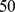 pixels.

**Figure 10:**
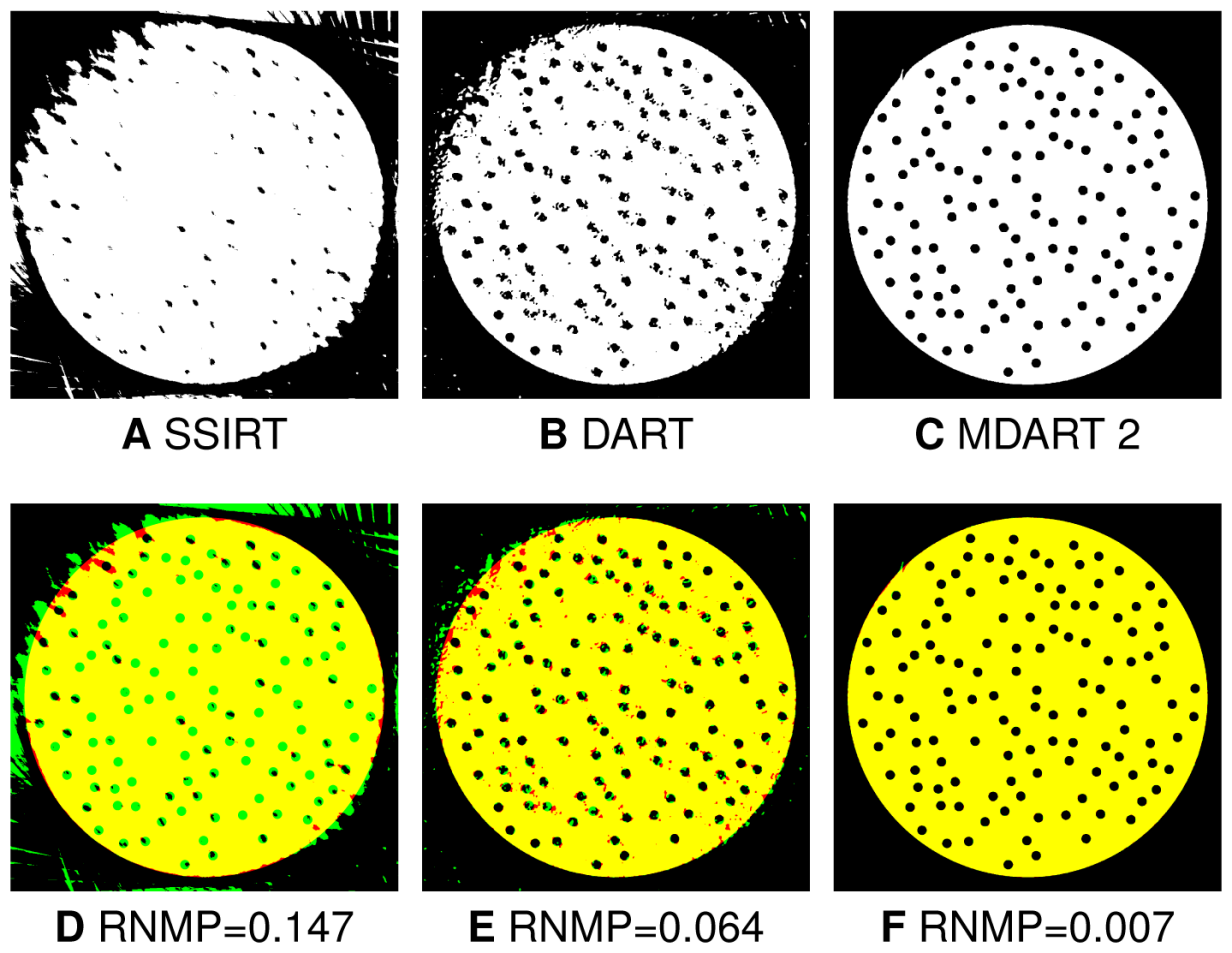
Reconstructions of the phantom with holes of radius 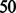 pixels. The reconstructions obtained after iterating for 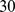 s with SSIRT (A), DART (B) and MDART 2 (C) using 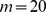 projections with 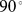 missing wedge together with the corresponding error images (D–F). Red and green in the error images correspond to misclassified background and object pixels, respectively, black and yellow represent correctly classified background and object pixels, respectively.

Figure 9 presents the obtained results after 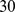 s iteration time, demonstrating the average RNMP over the phantoms with the holes of the particular size together with the standard errors (shown as shaded areas in the plots). Figure 10 shows the corresponding reconstructions of one of the phantoms with holes of radius 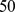 pixels calculated from 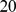 projections with 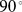 missing wedge. These plots demonstrate the ability of MDART to provide reconstructions of significantly higher quality compared to SSIRT and DART and to reduce missing wedge artefacts. The biggest gain compared to DART is achieved in the experiments with bigger missing wedge and smaller number of projections. The poor performance of MDART 4 on the phantoms with the hole radii of 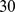 pixels is explained by the fact that on the coarsest reconstruction grid used by MDART 4 such holes have a radius of less than one pixel which complicates their detection with a discrete reconstruction algorithm. Note that for the holes of radius 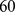 pixels or bigger MDART 4 shows the best results among all considered algorithms gaining from the use of coarser grids.

### Simulations with noise

**Figure 11:**
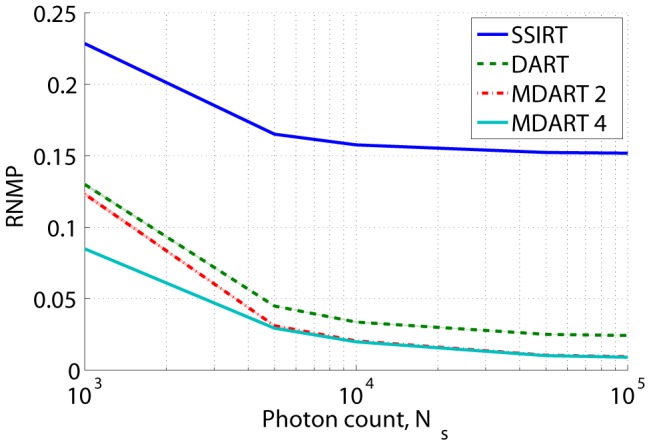
Results of the simulations with noise. RNMP as a function of the photon count for the reconstructions of the cylinder head phantom (Fig. 5B) from 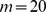 projections with noise. The iteration process was stopped after 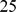 s.

In order to evaluate the proposed multiresolution approach in a more realistic situation, Poisson noise was added to one of the noiseless experiments. For the cylinder head phantom (Fig. 5B), 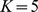 noisy sets of projection data were obtained for each noise level. For each noisy projection dataset the reconstructions were built. The mean values of 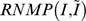 over these 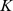 reconstructions after 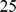 s iteration time are shown in Fig. 11, from which we see that the proposed method can outperform SSIRT and DART even in the presence of noise. This plot also demonstrates a slightly higher MDART 4 robustness against noise compared to MDART 2.

### Real experiments

The following experiments were conducted in order to demonstrate the performance of the proposed multiresolution approach on real data.

**Figure 12:**
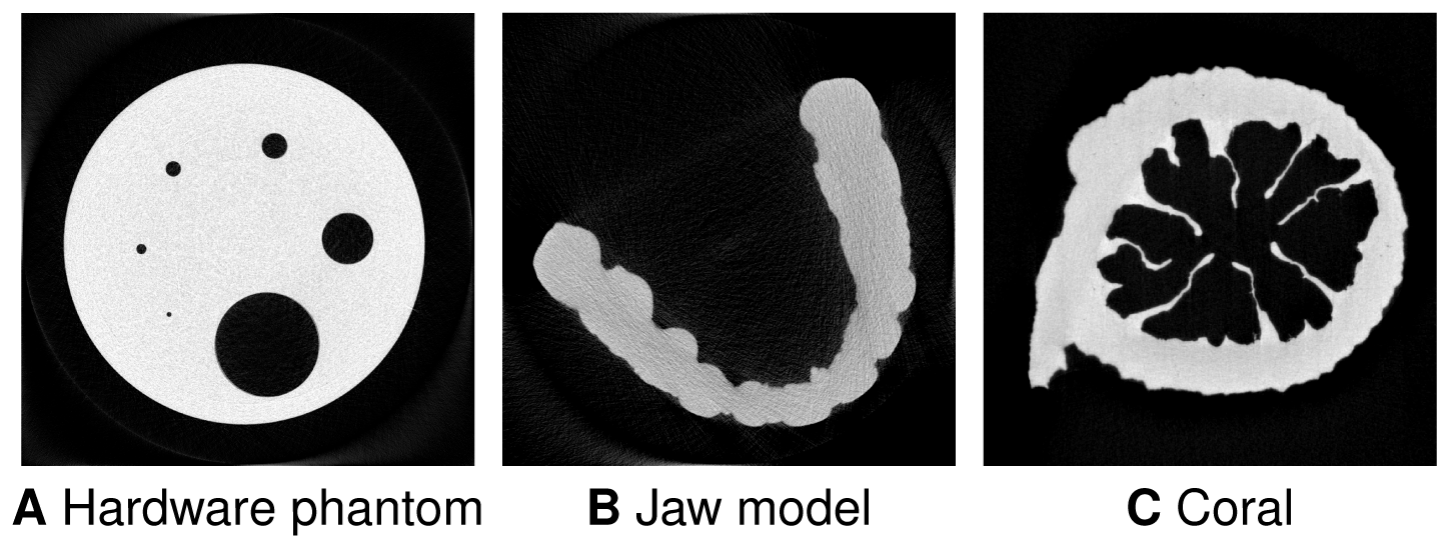
SIRT reconstructions of slices of the real datasets using all available projections. (A) The hardware phantom, 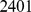 projections, (B) the jaw model, 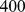 projections, (C) the coral, 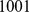 projections.

For the first experiment, a hardware phantom with a diameter of 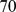 mm was scanned using the HECTOR micro-CT system developed by UGCT (the Ghent University Centre for X-ray Tomography, Belgium) in collaboration with X-Ray Engineering (XRE bvba, Ghent, Belgium) [22]. For this object, a full-angle cone-beam dataset was acquired containing 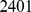 projections of 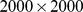 pixels, the X-ray tube voltage was 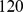 kV and the tube current was 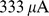. The source-detector distance was 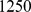 mm and the source-object distance was 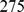 mm. One slice from this dataset was reconstructed with 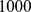 iterations of SIRT (Fig. 12A) on a 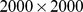 reconstruction grid with a pixel size of 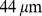.

In the second experiment, a gypsum jaw model was scanned using a desktop micro-CT system SkyScan-1172 (Bruker-MicroCT, Belgium). A full-angle cone-beam dataset consisting of 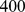 projections of 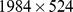 pixels was acquired, the X-ray tube voltage was 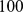 kV and the tube current was 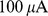. One slice from this dataset was reconstructed on a 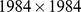 grid with a pixel size of 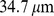 using 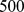 SIRT iterations (Fig. 12B).

Finally, a coral was scanned on the TOMCAT beamline [23] at the Swiss Light Source, Paul Scherrer Institut (Villigen, Switzerland). A full-angle parallel-beam dataset consisting of 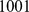 projections of 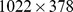 pixels was acquired, the beam energy was 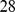 keV and the ring current was 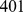 mA. One slice from this dataset was reconstructed on a 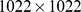 grid with a pixel size of 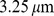 using 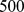 SIRT iterations (Fig. 12C).

The reconstructions using all available projections (Fig. 12) were segmented using the Otsu segmentation algorithm [24] and used as a ground truth in the following experiments. A number of 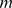 projections of the same slice were chosen from the corresponding original datasets to form datasets with limited angular ranges. These datasets were then reconstructed using the algorithms described above. Since true grey values to be used in DART and MDART were not known, these values were estimated as mean values in each segmentation class of the Otsu segmentation of the SIRT reconstructions shown in Fig. 12.

**Figure 13:**
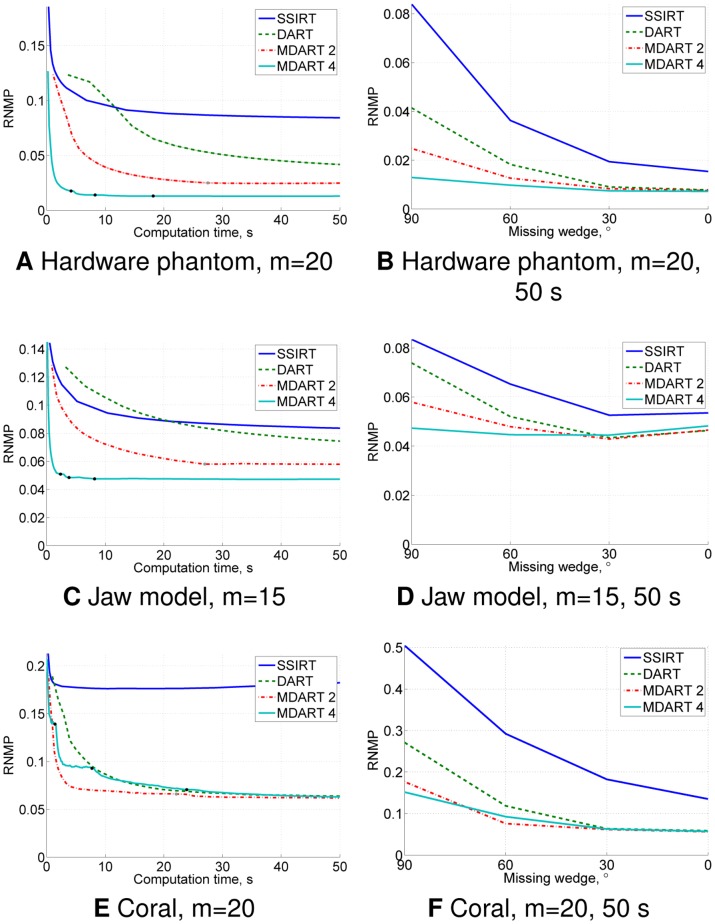
Results of the real data experiments. RNMP for the reconstructions of the real datasets (Fig. 12) as a function of the computation time from the data with the missing wedge (A, C, E) and as a function of the missing wedge after 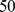 s iteration time (B, D, F). Missing wedge is 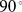 in (A) and (C) and 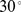 in (E). Black and grey points on the MDART curves (A, C, E) mark the moments of switching to a finer reconstruction grid.

**Figure 14:**
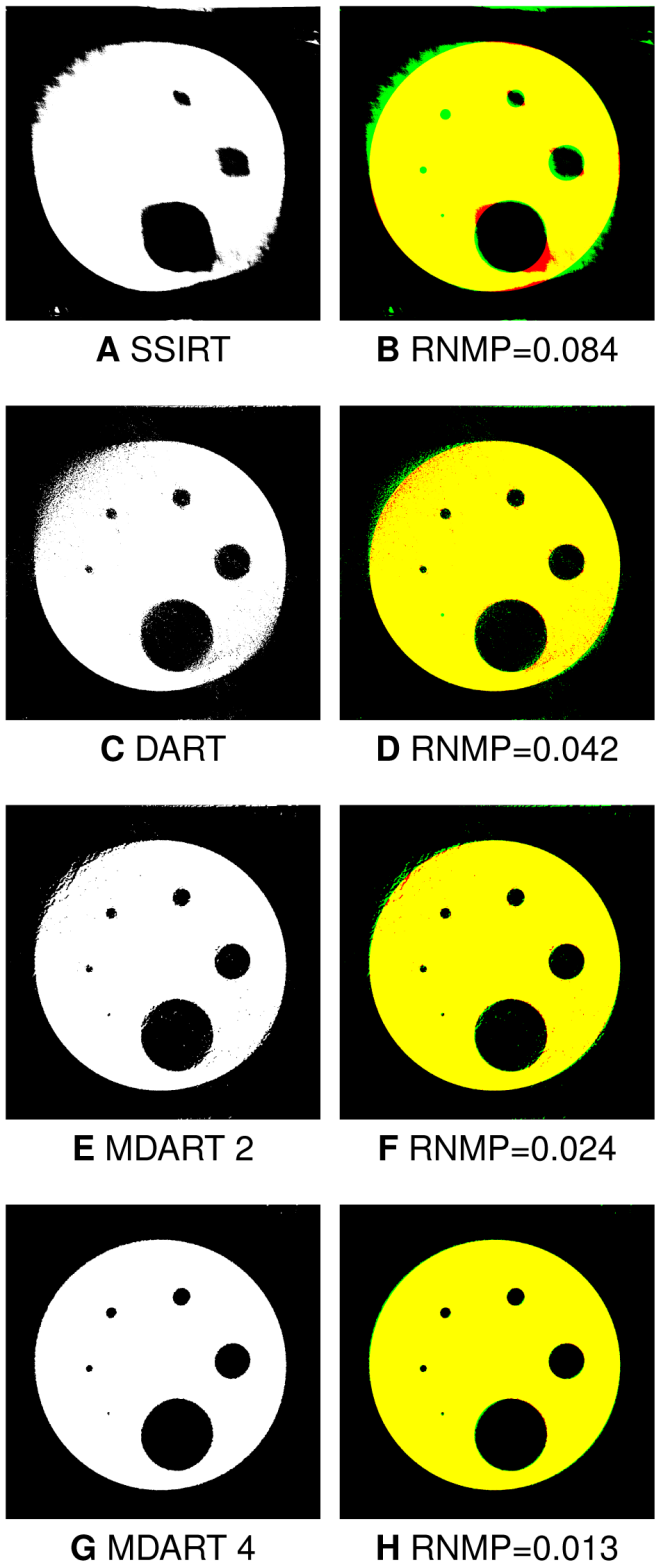
Reconstructions of the hardware phantom (Fig. 12A). The reconstructions obtained after iterating for 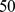 s with SSIRT (A), DART (C), MDART 2 (E) and MDART 4 (G) using 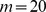 projections with 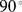 missing wedge together with the corresponding error images (B, D, F, H). Red and green in the error images correspond to misclassified background and object pixels, respectively, black and yellow represent correctly classified background and object pixels, respectively.

The obtained results are presented in Figs 13 and 14. Figures 13A, 13C and 13E demonstrate the ability of MDART to significantly speed up the reconstruction process and to yield more accurate results compared to SSIRT and DART. Figures 13B, 13D and 13F confirm that MDART suffers less from the missing wedge in the projection data than SSIRT and DART. The decreased performance of all methods on the jaw model dataset without the missing wedge compared to the dataset with the 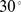 missing wedge (Fig. 13D) may be explained by the dependency of the reconstruction quality on the actual projection directions for some objects, especially if there are only a small number of projections used [25]. Moderate performance of MDART 4 on the coral dataset (Figs. 13E and 13F) compared to the performance of DART and MDART 2 is caused by the presence of very fine details in the object, which cannot be reconstructed on the coarsest reconstruction grid used by this algorithm. Examples of the reconstructions of the hardware phantom using 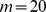 projections with 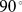 missing wedge shown in Fig. 14 suggest that the proposed approach, and MDART 4 in particular, can significantly reduce missing wedge artefacts and improve feature reconstruction for real objects. Therefore, experimental studies conform to the simulation experiments, showing the ability of the proposed approach to faster yield reconstructions of superior quality compared to those produced by SSIRT and DART for real datasets.

## Discussion

The proposed multiresolution DART algorithm starts a reconstruction on a coarse reconstruction grid and then uses the resampled resulting reconstruction as an initial point for a new reconstruction process on a finer grid, iteratively switching to the new grid until the target pixel size is reached. In our experiments, the next pixel size was always two times smaller than the current one. A certain variation in the pixel size changing strategy can have additional benefits in terms of computation time.

Experiments show that the proposed approach allows to create accurate reconstructions significantly faster than DART. Speed-up comes from the following two facts: iteration time decreases together with the number of pixels in the reconstruction and DART converges faster when starting from a better initial reconstruction. More accurate initial reconstruction results from the fact that use of the coarse grids makes the reconstruction problem less ill-posed decreasing the number of unknowns while preserving the number of equations. This is especially important in case when the limited number of projections is available or the projections were acquired from a limited angular range since the initial reconstruction calculated from such data can suffer from strong artefacts which sometimes slow down the convergence of conventional DART.

The choice of the starting pixel size has a significant influence on the performance of the proposed approach. On the one hand, the smaller the features present in the object, the smaller should be the starting pixel size. On the other hand, the bigger the starting pixel, the higher the potential for a speed-up and for robustness against noise. This trade-off should be made having a particular reconstruction problem in mind.

The proposed multiresolution approach can broaden the use of DART for large experimental datasets. It also allows to further decrease the number of projections required to obtain accurate reconstructions in a reasonable time.

## Conclusion

We proposed a multiresolution DART (MDART) algorithm for discrete tomography. This approach is based on the iterative use of a resampled reconstruction created on a coarse grid as a starting point for a subsequent reconstruction on a finer grid. Our experiments showed that MDART can lead to accurate reconstructions calculated in only a fraction of time compared to DART. The biggest improvement is reached for the datasets with a very small number of projections and acquired from a limited angular range. Reconstructions of the real datasets demonstrated an ability of MDART to significantly decrease the missing wedge artefacts and improve feature reconstruction in the object compared to the conventional DART algorithm being iterated for the same time.
